# What's new in peripheral T‐cell lymphomas

**DOI:** 10.1002/hon.2846

**Published:** 2021-06-09

**Authors:** Stefano Luminari, Tetiana Skrypets

**Affiliations:** ^1^ Surgical, Medical and Dental Morphological Sciences Related to Transplant, Oncology and Regenerative Medicine University of Modena and Reggio Emilia Reggio Emilia Italy; ^2^ Hematology Unit Azienda USL IRCCS of Reggio Emilia Reggio Emilia Italy; ^3^ PhD Programm in Clinical and Experimental Medicine University of Modena and Reggio Emilia Italy

**Keywords:** chemotherapy, novel agents, peripheral T‐cell lymphoma, prognostic factors

## Abstract

Peripheral T‐cell lymphomas (PTCLs) are a rare, heterogeneous group of hematological malignancies with extremely poor prognosis for almost all subtypes. The diverse clinicopathological features of PTCLs make accurate diagnosis, prognosis, and choice of optimal treatment strategies difficult. Moreover, the best therapeutic algorithms are still under debate due to the extrapolated approaches developed for B‐cell lymphomas and to the absence of few treatment protocol specifically developed for PTCLs. Some advances have been made with CD30 monoclonal antibody, mainly for anaplastic large‐cell lymphomas, with improvements in progression‐free survival and overall survival. Several new drugs are under evaluation in clinical trials, although not all the results are as encouraging as expected. In this review, we briefly present the most updated information on diagnosis, prognostication, and treatment strategies in PTCLs.

## 1 INTRODUCTION

Peripheral T‐cell lymphomas (PTCLs) comprise a heterogeneous subgroup of rare hematological malignancies originating from postthymic lymphocytes. From different available data, PTCLs account for approximately 5%–15% of all lymphomas in Western countries, with an incidence of 0.5–2 per 100,000 people per year.^1^, ^2^ Four main clinical subtypes have been identified: nodal, leukemic, disseminated, and cutaneous PTCLs. From a pathologic point of view, the most recent edition of the World Health Organization (WHO) Classification of lymphomas identifies around 30 subtypes of PTCLs, now also defined as mature T‐cell lymphomas (MTCLs), some of which are extremely rare. The most common MTCL subtypes are PTCL not otherwise specified (NOS), angioimmunoblastic T‐cell lymphoma (AITL), anaplastic large‐cell lymphoma (ALCL), and natural killer (NK)/T‐cell lymphoma (NKTCL).^1^, ^3^, ^4^ With the exception of cutaneous T‐cell lymphomas (CTCL), which are usually characterized by an indolent course, and of anaplastic lymphoma kinase (ALK) positive ALCL, all other MTCLs are associated with an aggressive course and poor outcomes, with 5‐year survival rates across subtypes of 30%.^3^

Improvement in the approach to MTCL is proceeding slowly, with advances in recent years seen in improving the classification of MTCL, patient management and prognostication, and treatment.

In the 2016 revision of the WHO of mature T‐cell neoplasms, nodal lymphomas of T follicular helper (TFH) cell origin were introduced. AITL is the most studied TFH subtype, but an additional 40% of cases of PTCL‐NOS have been shown to share some of the clinical and pathological features of the TFH phenotype, which requires the expression of at least two of three TFH‐related antigens, including PD1, CD10, BCL6, CXCL13, ICOS, SAP, and CCR5. Recurrent genetic abnormalities associated with TFH phenotype include mutations of in epigenetic modifiers (TET2, IDH2, DNMT3A), RHOA, and T‐cell receptor associated genes (PLCG1, CD28, VAV1, FYN).^3^, ^5^ Among the non‐TFH PTCL‐NOS, gene expression profiling and microRNA profiling studies have delineated two additional subgroups: those with an increased expression of GATA3 and those with an increased expression of TBX21.^3^, ^5^ The GATA3 group is associated with poor outcomes and has more loss or mutation of tumor suppressor genes including TP53, PTEN, PRDM1, and CDKN2A/B, and gains in STAT3, REL, and MYC oncogenes.^6^ The TBX21 group is enriched with alterations of genes involved in DNA methylation.^7^ These three groups are added to other previously characterized subtypes with specific cell of origin, which include ALCL, adult T‐cell leukemia/lymphoma, and gamma‐delta PTCL. The better characterization of the cell of origin in PTCL is advantageous in the classification of these lymphomas as it reduces the undefined basket of PTCL‐NOS and provides a strong rationale for determining the most effective therapies in these lymphomas. Immunomodulatory agents and epigenetic modifiers are more suitable for TFH subtypes, while phosphatidylinositol 3‐kinase (PI3K) inhibitors, hypomethylating agents, and Janus kinase/signal transducer and activator of transcription (JAK/STAT) inhibitors might find a better role in GATA3 or TBX21 subtypes.^8^

### 1.1 Prognosis and staging

In the last two decades, many studies have been conducted to identify and validate clinical and biological factors that can be used to predict the heterogenous outcome of PTCL patients. Several of these studies have confirmed that a poor Eastern Cooperative Oncology Group Scale of Performance Status score, extranodal involvement, advanced disease, bulky, Ki67, and a high lactate dehydrogenase rate correlate with shorter overall survival (OS).^9^, ^10^, ^11^ The International Prognostic Index (IPI) was formally validated in PTCL^1^, ^12^ but the lack of clearly defined risk groups prompted the search for PTCL‐specific prognostic indexes.^9^, ^11^ The Fondazione Italiana Linfomi defined the first Prognostic Index for PTCL‐NOS (PIT), which stratifies patients into four distinct groups; the PIT showed an independent correlation with OS. Subsequently, PIT was updated to modified PIT by replacing bone marrow involvement with Ki67 rate expression.^10^ A new model, the T‐cell score, has recently been defined by using the prospectively collected data registered in the T‐cell Project.^13^ More recently, novel prognostic indexes have been identified and validated for specific PTCL subtypes, including enteropathy‐associated T‐cell lymphoma (EATCL) and nasal‐type extranodal NKTCL. As shown in Table 1, all available prognostic indexes share the same structure of categorical scores based on simple laboratory and clinical features. Although all of these indexes have been formally validated, the accuracy of prognostication in PTCL remains suboptimal; thus, more prognostic studies that take into account novel biomarkers and novel prognostic features are warranted. Among recent advances, a better definition of response by means of ^18^F‐fluorodeoxyglucose positron emission tomography (FDG‐PET) may play an important role in PTCL management and decision making.^14^ PTCLs are listed among FDG‐avid diseases, and several studies have already confirmed the role of metabolic tumor volume and of interim and end‐of‐treatment FDG‐PET to predict outcomes.^15^, ^16^ Although promising, data regarding the role of metabolic response in PTCLs are very preliminary and thus need to be confirmed by larger studies.

**TABLE 1:** Prognostic models in PTCL

Variable	IPI	PIT	IPTCLP	mPIT	TCS	AITL	PINK	EPI
	International NHL Prognostic Factors Project (1993)	Gallamini et al. (2004)	Vose et al. (2008)	Went et al. (2006)	Federico et al. (2018)	Hong et al. (2018)	Kim et al. (2016)	de Baaij et al. (2015)
Age > 60	**X**	**X**	**X**	**X**		**X**		**X**
ECOG ≥ 1	**X**	**X**	**X**	**X**	**x**			**X**
LDH (abn. values)	**X**	**X**		**X**			**X**	**X**
Stage III–IV	**X**				**x**	**X**	**X**	**X**
ENS > 2	**X**					**X**		**X**
BM+		**X**						
Plt < 150 K/mmc			**X**			**X**		
S‐Alb					**x**			
Neutrophils					**x**			
Ki67 ≥ 80%				**X**				
Anemia (M < 13, F < 11g/dl)						**X**		
Serum IgA (>400 mg/dl)						**X**		
B symptoms							**X**	**X**
Regional lymph nodes							**X**	
PTCL subset	*All*	*PTCL‐NOS*	*PTCL‐NOS*	*PTCL‐NOS*	*PTCL‐NOS*	*AITL*	*NK‐TCL*	*EATL*

Abbreviations: AITL, angioimmunoblastic T‐cell lymphoma; BM, bone marrow; EATL, entheropathy‐associated T‐cell lymphoma; ENS, extranodal sites; EPI, EATL prognostic index; IPI, International Prognostic Index; ECOG, Eastern Cooperative Oncology Group; IPTCLP, International Peripheral T‐cell Lymphoma Project; LDH, lactate dehydrogenase; mPIT, modified Prognostic Index for T‐cell lymphoma; NKTCL, natural killer/T‐cell lymphoma; PINK, Prognostic Index of natural killer lymphoma; PLT, platelet count, PTCL‐NOS, peripheral T‐cell lymphoma not otherwise specified; S‐Alb, serum albumin; TCS, T‐cell score.

### 1.2 Treatment

The optimal management of patients with PTCL, which is disputed, is in any case limited to few options, all with unsatisfactory efficacy. None of the currently available recommendations are based on high‐quality evidence, and few well‐designed randomized clinical trials (RCTs) have been conducted to support therapeutic choices. The currently recommended treatment strategy for PTCLs derives mostly from B‐cell lymphoma treatment strategies, with the recommended use of an aggressive approach with anthracycline‐based polychemotherapy (i.e., CHOP or CHOEP) and with autologous stem cell transplant (autoSCT) to consolidate response to first‐line therapy or to manage relapsed patients.^17^

Regarding the role of anthracyclines in PTCL, while their role is still debated, anthracycline void regimens have so far failed to demonstrate their superiority to CHOP.^17^ Based on a recent meta‐analysis, the 5‐year OS achieved with this approach was 36.6%.^18^

Several attempts have been made to improve the poor results achieved with CHOP. These include the addition of novel agents and the intensification of therapy. Some clinical studies on etoposide intensification of standard CHOP have shown conflicting results. However, the addition of etoposide has shown better progression‐free survival (PFS), especially in patients with ALCL, in those with favorable risk factors, and in patients age ≤ 60 years.^12^, ^19^, ^20^, ^21^

The results of three randomized trials that evaluated the efficacy of adding a novel agent to the CHOP backbone are available.

One prospective trial combined alemtuzumab, an anti‐CD52 monoclonal antibody, with CHOP; it failed to show improved outcomes compared to chemotherapy alone.^22^ Another randomized trial compared standard CHOP with CHP (cyclophosphamide, vincristine, prednisone) combined with the anti‐CD30 antibody‐drug conjugate brentuximab vedotin (BV‐CHP; ECHELON‐2 trial).^23^ This trial, which enrolled 452 treatment naïve CD30‐positive PTCLs, demonstrated improved PFS and OS rates for the BV‐CHP combination.^23^ Most of the patients included in this trial (about 75%) had ALCL, with clearly positive results for this subtype. However, the scientific community was left without any clear demonstration of the efficacy of BV‐CHP in non‐ALCL CD30‐positive subtypes.

A third trial compared CHOP with CHOP + the histone deacetylase inhibitor romidepsin, showing promising single‐agent activity in relapsed refractory patients (ROCHOP trial).^24^ The ROCHOP RCT conducted by the LYSA group enrolled 421 patients with PTCL who were not planned to receive autoSCT or allogeneic SCT (alloSCT). The median PFS (mPFS) for patients in the experimental arm was 12 months (9–25.8), without significant difference compared to the reference arm (mPFS 10.2 months [7.4–13.2]; hazard ratio: 0.81; 95% confidence interval: 0.63–1.04). Although this study was not able to confirm the initial hypothesis of the superiority of Ro‐CHOP, the subgroup analysis seems to suggest that the novel combination acts differently in different PTCL subtypes, with relatively higher activity observed for AITLs.^24^

In summary, even if associated with unsatisfactory results, CHOP chemotherapy should still be considered as the reference therapy for most PTCL subtypes with the main exception of ALCL for which BV‐CHP is the preferred recommended option and of NKTCL. The use of CHOEP is supported by low quality of evidence but can be considered as a reasonable option in young and fit subjects with non‐ALCL PTCLs.

The use of high‐dose chemotherapy followed by autoSCT in first complete remission (CR1) is recommended by most of the available guidelines^17^, ^25^ (Table 2). Several groups have reported that achieving CR before autoSCT is a significant independent predictor of improved survival in patients with PTCL receiving upfront autoSCT.^26^, ^27^, ^28^ However, there have been no RCTs specifically designed to evaluate upfront autoSCT in comparison with observation in CR1 for PTCL.^29^, ^30^, ^31^ Several retrospective studies and prospective single‐arm phase II trials have reported encouraging results with this approach (Table 3). The largest prospective phase II study, published by the Nordic Group (NLG‐T‐01), included 160 patients with PTCLs; 72% of patients underwent autoSCT in first remission after six courses of CHOEP chemotherapy.^32^ All nodal PTCL subtypes were included, with the exception of A.

**TABLE 2:** ESMO and NCCN clinical practice guidelines for auto‐alloSCT in PTCLs

PTCLs subtype	Primary diagnosed PTCLs		Relapsed/refractory PTCLs	
	*ESMO*	*NCCN*	*ESMO*	*NCCN*
PTCL‐NOS	PR, CR, transplant eligible—autoSCT	Clinical trials, or ASCT, or observation if CR, or if PR—see rel/ref settings	PR, CR, transplant eligible—alloSCT (or ASCT)	PR, CR, transplant eligible—alloSCT (or ASCT)
AITL	PR, CR, transplant eligible—autoSCT	Clinical trials or ASCT or observation if CR, or if PR—see rel/ref settings	PR, CR, transplant eligible—alloSCT (or ASCT)	PR, CR, transplant eligible—alloSCT (or ASCT)
ALK‐negative ALCL	PR, CR, transplant eligible—autoSCT	Clinical trials, or ASCT or observation if CR, or if PR—see rel/ref settings	PR, CR, transplant eligible—alloSCT (or ASCT)	PR, CR, transplant eligible—alloSCT (or ASCT)
ALK‐positive ALCL	No further treatment, Or autoSCT if high‐risk profile	Only chemotherapy ± ISRT	PR, CR, transplant eligible—alloSCT (or ASCT)	PR, CR, transplant eligible—alloSCT (or ASCT)
EATL	autoSCT	Clinical trials, or ASCT, or observation if CR, or if PR—see rel/ref settings	PR, CR, transplant eligible—alloSCT (or ASCT)	PR, CR, transplant eligible—alloSCT (or ASCT)
HSTCL	ASCT or allo if donor available	CR or PR—preferred alloSCT	PR, CR, transplant eligible—alloSCT (or ASCT)	Preferred alloSCT if eligible
ENKTCL	ASCT	Stage IV if CR—allo or ASCT	PR, CR, transplant eligible—alloSCT (or ASCT)	AlloSCT (or ASCT)

Abbreviations: AITL angioimmunoblastic T‐cell lymphoma; ALCL, anaplastic large cell lymphoma; alloSCT, allogeneic stem cell transplant; ASCT, autologous stem cell transplant; CR, Complete remission; EATL, entheropathy‐associated T‐cell lymphoma; ENKTCL, entranodal T‐cell lymphoma; HSTCL, hepatosplenic T‐cell lymphoma; ISRT, involved site radiotherapy; PR, partial remission; PTCL, peripheral T‐cell lymphoma.

**TABLE 3:** Available prospective and retrospective studies of ASCT in PTCLs

Study	*N*	PTCLs subtype	Time of transplant	Response (%)	PFS (years)	OS (years)
Reimer et al. (2004)	83	39% PTCL‐NOS	Upfront	CR: 47	36% (4)	48% (4)
Prospective		16% ALCL (ALK‐negative)		PR: 24		
		33% AITL				
Corradini et al. (2006)	62	45% PTCL‐NOS	Upfront	CR: 56	EFS reported: 30% (12)	34% (2)
Prospective		30% ALCL (ALK‐positive)		PR: 16		
		16% AITL				
Rodriguez et al. (2007) Prospective	26	42% PTCL‐NOS	Upfront	CR: 65	53% (3)	86% (3)
		31% ALCL (ALK‐positive)		PR: 8		
		27% AITL				
Mercadal et al. (2008)	41	49% PTCL‐NOS	Upfront	CR: 49	30% (4)	39% (4)
Prospective		29% AITL		PR: 10		
		5% HSTCL				
		5% T/NK				
d'Amore et al. (2012)	160	39% PTCL‐NOS	Upfront	CR: 83	44% (5)	51% (5)
Prospective		19% ALCL (ALK‐negative)		PR: 31		
		19% AITL				
		13% EATL				
		4% panniculitis‐like				
		3% T/NK				
Fossard et al. (2018)	134	34% PTCL‐NOS	Upfront	CR: 75	46.3% (5)	59.2% (5)
Retrospective		23% ALCL (ALK‐negative)		PR: 25		
		43% AITL				
Roerden et al. (2019)	58	25.9% AITL	Upfront (40 pts)	CR: 75	Upfront ASCT	Upfront ASCT
		22.4% EATCL	Relapse/refractory (18 pts)	PR: 25	44% (5)	53% (5)
		20.7% PTCL‐NOS			ASCT in first relapse	ASCT in first relapse
Retrospective		19% ALCL (ALK‐negative)			60.6% (5)	77.4% (5)
		8.6% ALCL (ALK‐positive)				
		3.4% T/NK				
Park et al. (2019)	36	42% PTCL‐NOS	Upfront	CR: 63	44% (5)	51% (5)
Prospective		11% ALCL (ALK‐negative)		PR: 37		
		47% AITL				

Abbreviations: AITL, angioimmunoblastic T‐cell lymphoma; ALCL, anaplastic large cell lymphoma; ALK, anaplastic lymphoma kinase negative; ASCT, auto stem cell transplant; CR complete response; EATCL, enteropathy‐associated T‐cell lymphoma; EATL, entheropathy‐associated T‐cell lymphoma; EFS, event‐free survival; HSTCL, hepatosplenic T‐cell lymphomas; OS overall survival; PFS, progression‐free survival; PR, partial response; PTCLs, peripheral T‐cell lymphomas; PTCL‐NOS, peripheral T‐cell lymphomas not otherwise specified; T/NK, T‐cell lymphoma/natural killer.

#### 1.2.1 ALK‐positive ALCLs

One hundred thirty patients achieved CR (63%) or partial response (PR; 37%), and 115 (88.5%) underwent ASCT. Overall, the 5‐year OS and PFS for the intention‐to‐treat population were 51% and 44%, respectively. Considering subtype distribution, better outcomes were observed for ALK‐negative ALCL than for other subtypes.^32^ In a second study by Reimer et al.,^33^ 83 patients with PTCL were enrolled, with the exclusion of CTCL and of ALK‐positive ALCL. Fifty‐nine patients (71%) completed stem cell mobilization after CR (66%) or PR (34%) and 55 underwent autoSCT. The 3‐year OS was 48%.

Some recently published studies provide additional insights into the role of autoSCT in CR1. A real‐world data analysis from the Swedish Lymphoma Registry found prolonged OS and PFS for transplanted patients with PTCL‐NOS, AITL, ALK‐negative ALCL and EATCL after adjustment for confounding factors.^34^ However, the selection of non‐ASCT patients used as the control group may have been biased by early progressing patients after induction therapy. Another study was a large multicenter analysis conducted by the LYSA group. Among the 527 studied cases, a final cohort of 269 patients age less than 65 years with a CR or PR after induction chemotherapy was identified: 78 cases of PTCL‐NOS, 123 cases of AITL, and 68 cases of ALK‐negative ALCL. Overall, 81% were in CR and 19% in PR; 50% of the final cohort was allocated to autoSCT (134 patients). Neither the Cox multivariate model nor the propensity score analysis found any survival advantage in favor of autoSCT as a consolidation procedure for patients in response after induction therapy. Subgroup analyses did not reveal any further difference in terms of response status, disease stage, or risk category.^35^ Recently, Park et al.^36^ published their first report of the large prospective observational COMPLETE study, conducted by 56 U.S. academic centers. This paper described the outcomes of 119 patients who achieved CR after induction therapy, including 54 PTCL‐NOS, 35 AITL, and 30 ALK‐negative ALCL. Thirty‐six patients underwent autoSCT; patients with AITL had significantly improved OS and PFS but patients with other PTCL subtypes did not. Finally, an exploratory of the ECHELON‐2 trial was conducted, for the 82 patients with a declared intention to transplant out of the 177 patients randomized to BV‐CHP arm (ALK‐positive ALCL were excluded). SCT was in fact performed in 38 patients (27 ALK‐negative ALCL and 11 non‐ALCL patients), most of whom were from non‐Asian centers, suggesting regional practice differences. Despite the fact that the ECHELON‐2 study was not designed to evaluate the role of upfront consolidation with ASCT, numerical PFS estimates favored the use of consolidative SCT in patients with ALK‐negative ALCL and with non‐ALCL who achieved a CR at the end of induction after frontline BV‐CHP.^23^

Interpreting the results from these studies on the role of autoSCT consolidation is complicated by the diverse eligibility criteria adopted, the suboptimal rates of transplantation among PTCL subtypes, and the differing rates of CR before autoSCT. The decision to proceed to autoSCT in a subject with PTCL who responds to first‐line chemotherapy is difficult and should always be discussed with the individual patient. Researchers are strongly encouraged to run well‐designed clinical trials that adopt the same up‐to‐date criteria for response definition (i.e., FDG‐PET). These trials, which would necessarily require considerable international cooperation, would hopefully provide data by PTCL subtype.

alloSCT could be identified as alternative option to autoSCT as consolidation of CR1 patients. Schmitz et al.^37^ recently published data from the first randomized phase 3 trial of auto versus alloSCT as part of first‐line therapy in poor‐risk PTCLs excluding ALK‐positive ALCLs. The trial enrolled 18–60‐year‐old patients of all stages and IPI and was planned to detect an improvement of event‐free survival at 3 years from 35% achieved with autoSCT to 60% by alloSCT in the intent‐to‐treat population. After the enrollment of 104 patient randomization and recruitment was prematurely stopped because a planned interim analysis had shown that it was highly unlikely to meet the primary endpoint. The transplant‐related mortality observed contributed to this result. In conclusion, alloSCT cannot be recommended as consolidation therapy for CR1 PTCL patients due to the lack of evidence and because of it toxicity profile. The only exception to this general statement might be represented by hepatosplenic T‐cell lymphomas for whom a systematic review of 44 cases treated with alloSCT at first or second relapse demonstrated a 3‐year relapse‐free survival of 42% and OS of 56%.^38^

### 1.3 Relapsed/refractory disease

Approximately 70% of patients with PTCL are expected to develop relapsed or refractory disease after first‐line therapy.^39^, ^40^ A dismal outcome can be expected for these patients, with median OS of a few months, even for those who are able to proceed to salvage therapy.^39^ Among the options available, the effectiveness of autoSCT in relapsed disease is uncertain due to the frequent use of autoSCT in CR1 in eligible patients, and because the salvage therapies available for relapsed PTCLs have very limited activity, thereby further reducing the feasibility of an autoSCT program when planned. Available salvage regimens were previously developed as a first‐line strategies mostly for B‐cell malignancies, from them well‐known ICE (ifosfamide, carboplatin, etoposide), DHAP (high‐dose cytarabine, cisplatin, dexamethasone), GDP (gemcitabine, cisplatin, dexamethasone), and ESHAP (etoposide, cytarabine, cisplatin, and methylprednisolone). Despite the fact that these regimens were previously studied for aggressive lymphomas, due to the rarity of PTCLs a small number of patients were included without independent subset analysis.^41^, ^42^, ^43^, ^44^ Even if limited by a low power due, a subset analysis of the Canadian Cancer Trials Group LY.12 randomized phase 3 study was not able to confirm DHAP superiority over GDP in PTCLs.^45^ In relapsed/refractory PTCLs, alloSCT is also a feasible option in almost all subtypes after failing prior autoSCT.^17^, ^25^, ^46^ However, nonrelapse mortality varies from 8.2% to 40%.^47^, ^48^, ^49^ These scatter data suggest that it is necessary to carefully select possible candidates for alloSCT.

Several new agents have been tested in the relapsed refractory setting, and, while some have already received formal approval for clinical use, approval is not uniform across countries. These include the anti‐CD30 antibody‐drug conjugate BV, pralatrexate (approved in the United States only), and four histone deacetylase inhibitors (HDAC): romidepsin (United States only), belinostat (United States only), vorinostat (United States only), and chidamide (China only). Results achieved with these agents are very similar with CR rates of 10%–25% and with a median PFS of less than 1 year.

### 1.4 Novel agents

Pharmacology research is very active in PTCL, and therapeutic development is mainly driven by advances in the understanding of the biology of the disease (Table 4).

**TABLE 4:** Activity of novel agents from clinical trials in relapsed refractory PTCLs

Drug	PTCL subtype	No. of patients	Study phase	ORR; CR	Reference
Pralatrexate	PTCL	111	2	29%; 11%	O'Connor et al. (2011)
Romidepsin	PTCL	131	2	25%; 15%	Coiffier et al. (2012)
Brentuximab vedotin	CD30 + PTCL	34	2	41%; 24%	Horwitz et al. (2012); Pro et al. (2012)
	ALCL	58		86%; 57%	
Belinostat	PTCL	129	2	25.8%; 10.8%	O'Connor et al. (2015)
Bendamustine	PTCL	60	2	50%; 28%	Damal et al. (2013)
Mogamulizumab	CCR4 + PTCL/CTCL	29	1	34%; 17%	Ogura et al. (2014)
Lenalidomide	PTCL	39	2	26%; 8%	Tournishey et al. (2015)
Copanlisib	NHL	17	2	21%; 14%	Dreyling et al. (2017)
Cerdulatinib	PTCL	18	2a	43%	Horwitz et al. (2018)
Duvelisib	PTCL + CTCL	16	1	50%; 19%	Horwitz et al. (2018)
Alisertib	PTCL	271	3	33%; 18%	O'Connor et al. (2019)
Tipifarnib	AITL, CXCL12 + TCL	43	Interim analysis	45%	Witzig et al. (2019)
Pembrolizumab	PTCL	18		33%; 27%	Barta et al. (2019)
Panobinostat + bortezomib	PTCL	25	1	43%; 22%	Tan et al. (2015)
Gemcitabine + romidepin	PTCL	20	1	30%; 15%	Pellegrini et al. (2016)
Pralatrexate + romidepsin	TCL	14	1	71%; 29%	Amengual et al. (2018)
5‐Azacytidine + romidepsin	PTCL	31	1	73%; 55%	O'Connor et al. (2019)

Abbreviations: AITL, angioimmunoblastic T‐cell lymphoma; ALCL, anaplastic large‐cell lymphoma; CCR4, chemokine receptor‐4; CR, complete response; CTCL, cutaneous T‐cell lymphoma; CXCL12, C‐X‐C motif chemokine 12; NHL, non‐Hodgkin lymphoma; ORR, overall response rate; PTCL, peripheral T‐cell lymphoma; TCL, T‐cell lymphoma.

The frequent alteration of the epigenetic machinery in PTCL, mainly of the TFH phenotype, justifies strong rationale for the search of novel HDAC inhibitors and to test the efficacy of combining more than one epigenetic modifier. A recent phase 1 combination trial of 5‐azacitidine and romidepsin reported very interesting OR and CR rates of 73% and 55%, respectively.^50^

Inhibition of spleen tyrosine kinase (SYK) signaling and of PI3K pathways have also been investigated, with promising results from phase I and II studies. Cerdulatinib is an oral SYK, JAK1, JAK3, and Tyk2 inhibitor; in a phase IIa study on 41 PTCL patients, it was able to produce an overall response rate of 34%, with 27% CR rates.^51^ Among PI3K inhibitors, the oral duvelisib was used in a phase I study with 16 PTCL and 19 CTCL patients. The overall response rate was 50% for the PTCL patients and the median PFS was 8.4 months.^45^ The same agent has been evaluated in combination with romidepsin, showing greater activity in AITL and PTCL‐NOS (overall response rates 74% and 64%, respectively, CR rates 63% and 36%, respectively).^52^

A promising therapeutic strategy in PTCL is represented by targeting of tumor microenvironment. Blocking the PD1 interaction with its ligand is justified by the finding of an increased expression of PD‐L1 in both malignant and stromal cells of several PTCL subtypes. Indeed, some activity of anti‐PD1 agents in PTCL has been described by phase I studies,^53^ and more convincing results have been achieved with NKTCL. The use of PD1 blockers, however, has also been associated with cases of hyperprogression, thus making further clarification of PD1 inhibition in PTCL urgently needed.

Finally, cellular therapy based on the concept of chimeric antigen receptor T cells is also being developed for T‐cell lymphomas,^54^ as is the use of bispecific antibodies targeting both CD30 and CD16A.^55^

## 2 CONCLUSIONS

Management of PTCL patients continues to be a real challenge for hematologist–oncologists. The oversimplified approach that has been used for many years, replicating the rules of B‐cell lymphoma management, is clearly unsatisfactory and requires radical reassessment. New insights into the biology of the disease and a renewed interest on the part of the scientific community in the management of PTCL have led to the identification of new targets and to confirming the activity of new agents, which are now moving PTCLs into the era of targeted therapy. Moreover, taking into account the different biology and unique behavior of PTCL subtypes, each with a different response to therapy, has become indispensable; these differences result in more difficulties in interpreting the available data and in designing future trials. PTCL remains a challenging disease which requires massive international cooperation.

## CONFLICT OF INTERESTS

Luminari served as advisor for Roche, BMS, Jannsen, Regeneron, Genmab, Gilead. Skrypets has no conflict of interest to declare.
